# Protective factors for resilience in adolescence: analysis of a longitudinal dataset using the residuals approach

**DOI:** 10.1186/s13034-023-00687-8

**Published:** 2023-12-19

**Authors:** Jose Marquez, Louis Francis-Hew, Neil Humphrey

**Affiliations:** Manchester Institute of Education, University of Manchester, Manchester, M13 9PL UK

**Keywords:** Resilience, Adolescence, Wellbeing, Mental health, Life satisfaction, Adversity

## Abstract

**Introduction:**

The residuals approach, in which residual scores from regression models are used as a proxy for resilient functioning, offers great potential to increase understanding of resilience processes. However, its application in child and adolescent wellbeing research is limited to date. We use this approach to examine how adversity exposure impacts later wellbeing (life satisfaction, and internalising mental health difficulties) in the early-to-middle adolescence transition; whether gender and ethnic differences in resilience exist; which internal and external factors confer protective effects for resilience; and, whether the protective effect of these factors differs by gender and level of adversity exposure.

**Method:**

Secondary analysis of the #BeeWell longitudinal data set (N = 12,130 adolescents, aged 12/13 at T1 and 13/14 at T2, representative of Greater Manchester, England) was undertaken, using a series of linear regressions to establish adversity indices for later wellbeing, before assessing the protective effects of internal and external factors on resilience.

**Results:**

Multiple adversity factors (e.g., home material deprivation, sexuality discrimination, bullying) were found to impact later wellbeing. Girls and white adolescents presented lower levels of resilience than their peers. Internal psychological factors (self-esteem, emotional regulation, optimism) consistently conferred the strongest protective effects, but behavioural/activity factors (physical activity, sleep) also contributed to resilience. Among external factors, friendships and peer support were the most salient. Physical activity yielded stronger protective effects among boys (compared to girls). Effects of protective factors were stronger among those at lower (compared to higher) levels of adversity exposure.

**Conclusion:**

The residuals approach can make a considerable contribution to our understanding of the interplay between adversity exposure and access to protective factors in determining adolescent wellbeing outcomes. Moreover, its application provides clear implications for policy and practice in terms of prevention (of adversity exposure) and intervention (to facilitate resilience).

**Supplementary Information:**

The online version contains supplementary material available at 10.1186/s13034-023-00687-8.

## Introduction

Use of the term ‘resilience’ has become so ubiquitous that some have argued that it has become, “an empty word that can be filled with almost any meaning” [1]. However, a review of key resilience theories [2] offers some specificity, noting that all contain two core tenets. The first is the presence of *adversity* (sometimes also referred to as *risk, challenge*, or *stressors*). The second is the demonstration of *positive adaptation* (sometimes also referred to as positive *outcomes*, *adjustment*, *coping*, or *functioning*). Adversity refers to factors operating at multiple systemic levels that threaten adaptation or development [3], representing threat (the presence of harmful inputs in the environment, such as bullying) and/or deprivation (the absence of expected inputs from the environment, such as neglect) [4]. Critically, positive adaptation must be demonstrated *in spite of exposure to said adversity*. Thus, resilience can be defined as, “the capacity of a dynamic system to adapt successfully to disturbances that threaten system function, viability, or development” [5].

What engenders resilience? In her seminal work, Masten concludes that it is built through *ordinary* rather than *extra*ordinary processes (i.e., ‘ordinary magic’ [5]). In terms of how these processes operate, the adolescent resilience framework theorises that access to particular developmental assets moderates the relationship between exposure to adversity and developmental outcomes [6]. These assets are viewed as *protective factors* as their influence varies by levels of adversity exposure [3]. In both resilience theory [2] and the developmental assets framework [7], a distinction is drawn between those that are internal (e.g., self-esteem) and those that are external (e.g., parental support). Multiple reviews of the evidence base have identified a range of these protective factors that operate, like adversity factors, at multiple systemic levels [8–10]. None are particularly rare or special qualities, offering support for the ordinary magic thesis [9].

In the current study, we focus on adolescent resilience, with specific reference to wellbeing outcomes. Wellbeing has traditionally been conceptualized through two main theoretical lenses. First, in the hedonic/subjective framework, it comprises an affective component (positive and negative affect), and a cognitive component (life satisfaction, LS), and emphasizes ‘feeling good’ [11]. Second, in the eudaimonic/psychological framework, wellbeing is defined in terms of autonomy, purpose in life, environmental mastery, personal growth, optimism, self-acceptance, and/or positive relations with others, and emphasizes ‘flourishing’ [12]. Broader conceptualisations of wellbeing include symptoms of mental health (MH) difficulties and/or use the terms wellbeing and MH interchangeably (e.g., [13, 14]).

Taking these different perspectives into consideration, we operationalise wellbeing by focusing on outcomes reflecting subjective wellbeing (LS) and symptoms of MH difficulties (internalising symptoms). Both have demonstrable utility and salience in adolescence (and beyond). For example, internalising MH difficulties are particularly prevalent during the teenage years [15], impair quality of life, and are concurrently and prospectively associated with academic attainment and a range of other important outcomes [16]. LS is the most commonly used indicator of subjective wellbeing [17], declines during adolescence [18, 19], and is widely considered to contribute valuable information over and above more direct, health-related measures (such as internalising symptoms) [20].

### Measuring resilience: the residuals approach

As a concept that is inferred rather than observed, measuring resilience is challenging; a variety of approaches are evident in the current literature, none of which are considered to offer a ‘gold standard’ [21–23]. However, one very promising and innovative method that has emerged in recent years is the so-called ‘residuals’ approach. The basic premise is that a metric of resilience can be derived using standardised residual scores from models in which an outcome representing adjustment on a construct of interest (e.g., internalising symptoms) is regressed onto a series of adversity factors (e.g., exposure to discrimination) [23]. The standardised residual score for such a model represents the difference between *actual* adjustment and adjustment *predicted* by adversity. This enables us to identify those who are doing better than would be expected based on their exposure to adversity (i.e., those who are ‘resilient’) [22].

The residuals method is particularly attractive as it quantifies resilience using a continuous, meaningful metric from vulnerability (negative residual scores that fall below the fitted regression line) to resilience (positive residual scores that fall above the fitted regression line). It also enables analyses that offer specificity in terms of underpinning protective processes (i.e., the extent to which a putative protective factor predicts later resilience, and whether this varies by levels of adversity exposure) [22]. However, its application to date in the study of child and adolescent resilience is limited. The research which has been undertaken can be divided into studies that adopt a population health perspective (thereby drawing on large, representative cohort datasets), and studies that take a clinical approach (thereby drawing on targeted/indicated datasets, such as offspring of depressed parents [24], trauma-exposed adolescents [25], or those considered to be at-risk [26]. The current study is an example of the population health approach; accordingly, we review analogous research below.

Focusing on familial adversities, Miller-Lewis et al. [22] analysis of longitudinal data from Australian children used the residuals method to identify both internal (e.g., self-concept) and external (e.g., child-teacher relationships) factors associated with greater MH resilience in a two-year period between pre-school and school entry, while also noting those whose effects were amplified under conditions of high adversity (e.g., self-control). Also focusing on familial adverisities, but instead working with adolescents, Van Harmelen et al. [27] analysis of 1-year longitudinal data identified friendship support as a significant positive predictor, and (somewhat surprisingly) family support as a *negative* predictor, of later resilient functioning. Finally, Cahill et al. [23] drew on a range of factors spanning parent/familial (e.g., parental mental health problems), peer group (e.g., bullying), and neighbourhood (e.g., low neighbourhood satisfaction) adversities experienced during adolescence to examine MH resilience during emerging adulthood. The authors identified a number of protective factors, including self-esteem, positive sibling relationships, good temperament, and positive perceptions of school.

It is important to note that the interplay between adversity exposure and access to protective factors in determining adolescent wellbeing outcomes is not expected to be uniform across the adolescent population. First, we might reasonably expect effects to vary across gender, resulting from socialisation and intensification effects in relation to, for example, expectations and values pertaining to putative protective factors such as friendships and social support [28, 29]. Second, the social-ecological systems within which development occurs are not culturally neutral, and it has long been theorised that the mechanisms that support adjustment to adversity are, at least in part, culturally specific [30, 31], meaning we might reasonably expect ethnicity to moderate resilience. Finally, while protective factors are, by definition, those that confer advantage in the presence of adversity (operationalised in residuals studies through their association with the residuals score), it stands to reason that variability in levels of adversity exposure may moderate the magnitude of their effects, with some factors more (or less) potent in the context of increasing adversity [5].

However, findings to date are inconsistent and/or require further investigation. Thus, with regard to gender, while some adolescent studies using the residuals approach have identified gender differences in resilience [27, 32], some have not [23], while others have found that the informant (i.e., parent-report vs teacher-report of the outcome in question) may determine the presence or absence of any disparities [22]. In the only adolescent residuals study to our knowledge that has focused on ethnicity, Cahill et al. [22] found no evidence of moderation. However, these authors opted for a binary approach to coding ethnicity data (i.e., White vs. ethnic minority), which may have masked important differences across discrete ethnic minority groups. Finally, with regard to levels of adversity, the aforementioned study by Miller-Lewis et al. [23] found that self-control during preschool provided greater protection to children exposed to higher levels of familial adversity, but other candidate protective factors, such as parent–child relationships, did not.

### The current study

In the current study, we use the residuals approach to identify internal and external protective factors for adolescent resilience at multiple system levels. We build upon the above evidence base and extend it in a number of important ways. First, we focus on the transition between early- and mid-adolescence (age 12–14), which immediately precedes the peak age-of-onset for lifetime cases of MH difficulties [33], thereby enabling insights into protective processes during a period of particular vulnerability. Second, we use data from a large sample collected after the Covid-19 pandemic (Autumn 2021 onwards). This is particularly critical given its impact on young people’s MH [34], and consequent implications for the nature and extent of adversity exposure and access to protective factors [35]. Third, we use a longitudinal dataset. Doing so enables temporal precedence to be established (i.e., adversity exposure and access to protective factors preceding wellbeing outcomes). This is not possible in cross-sectional research, which remains predominant in the study of adolescent resilience research [8].

Fourth, in alignment with the multi-systemic approach to resilience [9], we consider a broader range of adversity and protective factors than has been typical in applications of the residuals approach in the study of child and adolescent resilience to date (e.g., [22, 23]), drawing on theory and evidence to support our selection and classification approach. A case in point is sleep hygiene. Like many other candidate factors, sleep could potentially be conceptualised as an adversity factor (i.e. poor sleep hygiene) or a protective factor (i.e. good sleep hygiene) [3]. In this study, we consider it as a protective factor, similarly to physical activity, given theoretical and empirical support indicating that such health behaviours could promote resilience during adolescence [36, 37], and their obvious tractability, which provides avenues for intervention.

Fifth, we assess convergences and divergences in the influence of these factors on two important wellbeing outcomes: LS and internalising MH difficulties. Finally, we also consider socio-demographic differences in resilience and protective factors. The following research questions (RQs) drive our inquiry:

1. How does adversity exposure impact later wellbeing during the transition from early to middle adolescence?
2. Are there gender and ethnic differences in resilience in this period?
3. Which internal (sleep hygiene, physical activity, self-esteem, emotion regulation, and optimism), and external (parents/carer support, friendships and social support, and school staff support) factors confer protective effects for adolescent resilience?
4. Does the protective effect of these factors differ by gender and level of adversity exposure?

Our intended contribution to the broader field of adolescent mental health and wellbeing research in addressing these important questions is to provide insights into the often overlooked yet critical aspect of resilience processes in this population. The residuals approach not only sheds light on the impact of adversity exposure on later wellbeing, but also offers a novel lens through which to examine the protective effects of internal and external factors. By providing a nuanced exploration of the interplay between adversity exposure and access to protective factors in determining adolescent wellbeing outcomes, we hope to pave the way for informed policy and intervention strategies that promote adolescent wellbeing by addressing adversity exposure and fostering resilience factors.

## Methods

### Sample

Our secondary analysis draws on the first (T1) and second (T2) annual data points of the #BeeWell study in Greater Manchester [38]. #BeeWell’s hybrid population cohort study design includes a truncated longitudinal study in which participants are surveyed with annual data points from age 12–15 (e.g. from Year 8 to Year 9 to Year 10 of secondary school; Sample 1) and a repeated cross-sectional study comprising annual data points for participants aged 14–15 (e.g. those in Year 10 of secondary school at a given data point; Sample 2). We consider all adolescents from the Sample 1 at T1 (2021) (Year 8, aged 12–13, N = 20,241) who took the survey in T2 (2022) when they were Year 9, aged 13–14 (N = 12,130).

Given the attrition rate observed between, we compared complete (T1 and T2) and incomplete (T1 only) cases (see Additional file 1: Table S1). Differences were small and trivial for socio-demographic variables (e.g., gender, ethnicity), candidate protective factors (e.g., physical activity), and adversity factors (e.g., bullying victimisation). Notable exceptions to this were the two socio-economic adversity indicators, where larger differences were observed (c.7% higher among incomplete cases), a point which we return to in the Discussion section.

Within our analytic sample, individual-level data were available on a wide range of wellbeing domains/indicators and drivers, in addition to multiple individual characteristics, from which we derive the adversity factors, wellbeing outcomes, candidate protective factors, and co-variates outlined in Table 1.

**Table 1:** Characteristics of the Sample (12,130 12–14-Year-Old Adolescents) and Descriptive Information of Study Variables

Variable	Value		Description	Source
Adversity variables (T1)	n	%		
Bullying Victimisation (9.05% Missing Data)			Responded ‘quite a lot’ or more in at least 1 of the 3 #BeeWell items on bullying: physically bullied at school, bullied in other ways at school, and cyber-bullied Timing of adversity exposure: in the 6 months leading up to survey response in Autumn 2021 (T1)	Items adapted from the Understanding Society Youth Questionnaire [39] and the Health Behaviours in School-Aged Children survey [40]
*Yes*	1876	(17.01%)		
*No*	9156	(82.99%)		
Free School Meal Eligibility (2.80% Missing Data)			Eligible for free school meals in the previous 6 years Timing of adversity exposure: age from 6/7 to 12/13	Linked administrative data provided by the 10 Greater Manchester Local Authorities
*Yes*	2835	(24.05%)		
*No*	8955	(75.95%)		
Neighbourhood Socio-Economic Disadvantage (3.80% Missing Data)			Lives in a neighbourhood that belongs to the most deprived decile in the 2019 Index of Mulitple Deprivation (IMD) Time of adversity exposure: between 0 and 12/13 years	Linked administrative data provided by the 10 Greater Manchester Local Authorities
*Yes*	2814	(24.12%)		
*No*	8855	(75.88%)		
Home Material Deprivation (8.14% Missing Data)			Score of less than 5 on the 0–10 #BeeWell item on home material deprivation ("How happy are you with the things that you have (like money and the things that you own)?"; 0 = very unhappy, 10 = very happy) Timing of adversity exposure: Autumn 2021 (T1)	Item from the Good Childhood Index [41]
*Yes*	368	(3.30%)		
*No*	10,755	(96.70%)		
Racial Discrimination (8.91% Missing Data)			Did not report 'never' to the #BeeWell item on racial discrimination ("How often do people make you feel bad because of: your race, skin colour or where you were born") Timing of adversity exposure: Autumn 2021 (T1)	Item adapted from the Determinants of Adolescent Social Wellbeing and Health Study survey and the Measuring Discrimination Resource [42]
*Yes*	3022	(27.35%)		
*No*	8027	(72.65%)		
Gender Discrimination (9.33% Missing Data)			Did not report 'never' to the #BeeWell item on gender discrimination ("How often do people make you feel bad because of: your gender") Timing of adversity exposure: Autumn 2021 (T1)	Item adapted from the Determinants of Adolescent Social Wellbeing and Health Study survey and the Measuring Discrimination Resource [42]
*Yes*	2321	(21.10%)		
*No*	8677	(78.90%)		
Sexuality Discrimination (10.66% Missing Data)			Did not report 'never' to the #BeeWell item on sexuality discrimination ("How often do people make you feel bad because of: your sexual orientation") Timing of adversity exposure: Autumn 2021 (T1)	Item adapted from the Determinants of Adolescent Social Wellbeing and Health Study survey and the Measuring Discrimination Resource [42]
*Yes*	1761	(16.16%)		
*No*	9137	(83.84%)		
Disability Discrimination (9.77% Missing Data)			Did not report 'never' to the #BeeWell item on disability discrimination ("How often do people make you feel bad because of: your disability") Timing of adversity exposure: Autumn 2021 (T1)	Item adapted from the Determinants of Adolescent Social Wellbeing and Health Study survey and the Measuring Discrimination Resource [42]
*Yes*	1310	(11.97%)		
*No*	9635	(88.03%)		
Religious Discrimination (9.51% Missing Data)			Did not report 'never' to the #BeeWell item on religious discrimination ("How often do people make you feel bad because of: your religion/faith") Timing of adversity exposure: Autumn 2021 (T1)	Item adapted from the Determinants of Adolescent Social Wellbeing and Health Study survey and the Measuring Discrimination Resource [42]
*Yes*	1801	(16.41%)		
*No*	9176	(83.59%)		
Special Educational Needs (1.45% Missing Data)			Identified by the local authority as having special educational needs or disability Time of adversity exposure: age 4 to 12/13	Linked administrative data provided by the 10 Greater Manchester Local Authorities
*Yes*	1,759	(14.71%)		
*No*	10,195	(85.29%)		
Feeling Unsafe in Local Area (7.86% Missing Data)			Responded 'disagree'/'strongly disagree' to the #BeeWell local environment item on safety in the local area ('I feel safe in the area where I live') Timing of adversity exposure: Autumn 2021 (T1)	Item adapted from the Health Behaviours in School-Aged Children survey [40]
*Yes*	686	(6.14%)		
*No*	10,490	(93.86%)		
Unhappy with Home Environment (7.94% Missing Data)			Score of less than 5 on the 0–10 #BeeWell item on home environment ("How happy are you with the home that you live in?"; 0 = very unhappy, 10 = very happy) Timing of adversity exposure: Autumn 2021 (T1)	Item from the Good Childhood Index [41]
*Yes*	587	(5.26%)		
*No*	10,580	(94.74%)		
Caregiving Responsibilities (9.20% Missing Data)			Responded 'yes' to the #BeeWell item on caregiving responsibility ("Do you care for a family member who has an illness, disability, mental health condition, or drug/alcohol dependency?") Timing of adversity exposure: Autumn 2021 (T1)	Item from the Wellbeing Measurement Framework [43]
*Yes*	5275	(47.98%)		
*No*	5739	(52.11%)		
Suboptimal Physical Health (7.21% Missing Data)			Responded 'fair’ or ‘poor’ to the #BeeWell item on physical health ("In general would you say your physical health is") Timing of adversity exposure: Autumn 2021 (T1)	Item from the Understanding Society Youth Questionnaire [39]
*Yes*	1528	(13.58%)		
*N*	9727	(86.42%)		

Wellbeing outcome variables (T2)	Mean	S.D.		
LS—Overall Life Satisfaction (8.00% Missing Data)	6.75	(2.41)	#BeeWell's 0–10 life satisfaction scale ("Overall, how satisfied are you with your life nowadays?"; 0 = Not at all, 10 = Completely)	Item from the Office for National Statistics’ personal wellbeing item bank [44]
MH—Internalising Symptoms (10.52% Missing Data)	12.40	(4.83)	0–20 scale derived the 10 #BeeWell negative affect items	Items from the emotional difficulties subscale of the Me and My Feelings questionnaire [45]

Protective factors (T1)	n	%		
Sleep hygiene (7.86% Missing Data)			#BeeWell's item on sleep hygiene: "Is the amount of sleep you normally get enough for you to feel awake and concentrate on your school work during the day?" (Yes/No)	Item from the Health Behaviours in School-Aged Children survey [40]
*Yes*	7351	(65.77%)		
*No*	3826	(34.23%)		
	Mean	S.D.		
Physical activity	354.89	(233.87)	Scale from 0 to 840 min (0, 30, 60, 90,…840) derived from two #BeeWell items: (i) how many days in a usual week they are physically active; and, (ii) on those days, how long on average they spend being physically active. The resultant data are combined to produce a continuous estimate of weekly physical activity	Items adapted from the Health Behaviours in School-Aged Children survey [40]
Self-Esteem (12.14% Missing Data)	14.84	(3.35)	5–10 scale derived from the six #BeeWell items on self-esteem	Items from the Rosenberg Self-Esteem Scale [46, 47]
Emotional Regulation (14.04% Missing Data)	24.39	(6.88)	6–42 scale derived from the six #BeeWell items on emotional regulation	Items from the Trait Emotional Intelligence Questionnaire – Adolescent Short Form [48]
Optimism	12.04	(3.72)	4–20 scale derived from the four #BeeWell items on optimism	Items from the optimism subscale of the Engagement, Perseverance, Optimism, Connectedness, Happiness (EPOCH) measure of adolescent wellbeing [49]
School Staff Support (9.93% Missing Data)	15.22	(3.94)	4–20 scale derived from the 4 #BeeWell items on relationships with school staff	Items from the school connection subscale of the Student Resilience Survey [50]
Friendships and Social Support (9.90% Missing Data)	15.60	(3.55)	4–20 scale derived from the four #BeeWell items on friendships and social support	Items from the Child and Youth Resilience Measure [51]
Parents/Carers Support (9.04% Missing Data)	17.43	(3.13)	4–20 scale derived from the four #BeeWell items on relationships with parents/carers	Items from the family connection subscale of the Student Resilience Survey [50]
Covariates (T1)				
Gender (No Missing Data)			Identified by the local authority as male or female	Linked administrative data provided by the 10 Greater Manchester Local Authorities
*Male*	6225	(48.65%)		
*Female*	5898	(51.35%)		
Ethnicity (2.46% Missing Data)			Categorised by the local authority as belonging to one of these ethnic groups	Linked administrative data provided by the 10 Greater Manchester Local Authorities
*White*	7741	(65.43%)		
*Black*	623	(5.27%)		
*Asian*	2091	(17.67%)		
*Chinese*	115	(0.97%)		
*Any Other Ethnic Group*	291	(2.46%)		
*Mixed Race*	701	(5.93%)		
*Unclassified*	269	(2.27%)		

n = number of observations; *S.D.* Standard Deviation. T1 indicates that the data were collected in the first year of the #BeeWell study (Autumn 2021), when participants were 12–13 years old. T2 indicates that the data were collected in the second year of the #BeeWell study (Autumn 2022), when participants were 13–14 years old

### Measures

Study variables are detailed in Table 1. Data pertaining to adversity and protective factors and sociodemographic covariates were drawn from the T1 survey and linked administrative data provided by the 10 Greater Manchester Local Authorities. LS and MH data from T2 were used to estimate the residuals in the first step of our analysis, and to study protective factors in the second step (see Analytical Strategy). The correlation between the study variables is presented in Table 2.

**Table 2:** Correlations Between the Study Variables

Variables	1	2	3	4	5	6	7	8	9	10	11	12	13	14	15	16	17	18	19	20	21	22	23	24
(1) LS—Life Satisfaction (T2)	1																							
(2) MH—Internalising Symptoms (T2)	− 0.593	1																						
(3) Bullying Victimisation (Adv.) (T1)	− 0.201	0.252	1																					
(4) Free School Meal Eligibility (Adv.) (T1)	− 0.069	0.034	0.050	1																				
(5) Neighbourhood Socio-Economic Disadvantage (Adv.) (T1)	− 0.034	0.005	0.011	0.229	1																			
(6) Home Material Deprivation (Adv.) (T1)	− 0.141	0.098	0.118	0.056	0.030	1																		
(7) Racial Discrimination (Adv.) (T1)	− 0.144	0.104	0.122	0.059	0.094	0.080	1																	
(8) Gender Discrimination (Adv.) (T1)	− 0.237	0.280	0.218	0.023	− 0.005	0.116	0.316	1																
(9) Sexuality Discrimination (Adv.) (T1)	− 0.234	0.284	0.247	0.042	0.008	0.103	0.257	0.543	1															
(10) Disability Discrimination (Adv.) (T1)	− 0.132	0.145	0.216	0.051	0.005	0.093	0.236	0.297	0.328	1														
(11) Religious Discrimination (Adv.) (T1)	− 0.097	0.081	0.107	0.017	0.058	0.079	0.450	0.291	0.241	0.249	1													
(12) Special Educational Needs (Adv.) (T1)	− 0.019	0.027	0.080	0.104	0.043	0.042	− 0.014	0.011	0.036	0.240	− 0.003	1												
(13) Feeling Unsafe in Local Area (Adv.) (T1)	− 0.095	0.086	0.100	0.049	0.059	0.124	0.046	0.065	0.060	0.069	0.046	0.034	1											
(14) Unhappy with Home Environment (Adv.) (T1)	− 0.178	0.143	0.132	0.072	0.040	0.373	0.082	0.143	0.133	0.101	0.077	0.043	0.181	1										
(15) Caregiving Responsibilities (Adv.) (T1)	− 0.018	− 0.008	0.078	0.092	0.063	0.013	0.051	0.008	0.021	0.093	0.034	0.084	0.036	0.018	1									
(16) Suboptimal Physical Health (Adv.) (T1)	− 0.234	0.237	0.183	0.076	0.043	0.155	0.101	0.153	0.169	0.119	0.069	0.034	0.111	0.203	0.037	1								
(17) Sleep Hygiene (P.F.) (T1)	0.278	− 0.291	− 0.175	− 0.050	− 0.017	− 0.121	− 0.102	− 0.196	− 0.194	− 0.106	− 0.063	− 0.026	− 0.114	− 0.145	− 0.050	− 0.277	1							
(18) Physical Activity (P.F.) (T1)	0.134	− 0.155	− 0.039	− 0.060	− 0.051	− 0.049	− 0.031	− 0.072	− 0.107	− 0.028	− 0.049	− 0.043	− 0.007	− 0.075	0.011	− 0.154	0.090	1						
(19) Self-Esteem (P.F.) (T1)	0.407	− 0.416	− 0.255	− 0.053	− 0.022	− 0.163	− 0.112	− 0.270	− 0.282	− 0.146	− 0.080	− 0.031	− 0.130	− 0.213	− 0.040	− 0.358	0.358	0.194	1					
(20) Emotional Regulation (P.F.) (T1)	0.370	− 0.421	− 0.264	− 0.050	− 0.008	− 0.107	− 0.125	− 0.255	− 0.265	− 0.172	− 0.090	− 0.063	− 0.105	− 0.161	− 0.077	− 0.280	0.367	0.105	0.509	1				
(21) Optimism (P.F.) (T1)	0.375	− 0.361	− 0.179	− 0.046	0.011	− 0.142	− 0.088	− 0.227	− 0.240	− 0.122	− 0.059	− 0.015	− 0.080	− 0.179	0.008	− 0.308	0.346	0.190	0.569	0.444	1			
(22) School Staff Support (P.F.) (T1)	0.256	− 0.179	− 0.138	− 0.047	0.005	− 0.156	− 0.144	− 0.162	− 0.142	− 0.110	− 0.106	− 0.006	− 0.086	− 0.166	− 0.026	− 0.212	0.268	0.086	0.340	0.270	0.389	1		
(23) Friendships and Social Support (P.F.) (T1)	0.294	− 0.294	− 0.295	− 0.053	− 0.023	− 0.171	− 0.141	− 0.222	− 0.231	− 0.191	− 0.125	− 0.070	− 0.108	− 0.192	− 0.032	− 0.268	0.263	0.165	0.446	0.351	0.438	0.368	1	
(24) Family Support (P.F.) (T1)	0.273	− 0.200	− 0.162	− 0.100	− 0.037	− 0.216	− 0.149	− 0.212	− 0.240	− 0.178	− 0.116	− 0.087	− 0.121	− 0.270	− 0.079	− 0.232	0.271	0.114	0.354	0.278	0.372	0.425	0.392	1

*Adv.* Adversity, *P.F.* protective factor. T1 indicates that the data were collected in the first year of the #BeeWell study (Autumn 2021), when participants were 12–13 years old. T2 indicates that the data were collected in the second year of the #BeeWell study (Autumn 2022), when participants were 13–14 years old

#### Adversity (T1)

14 adversity variables were considered: bullying victimisation; free school meal eligibility (FSM); neighbourhood socio-economic disadvantage; home material deprivation; racial discrimination; gender discrimination; sexuality discrimination; disability discrimination; religious discrimination; special educational needs (SEN); feeling unsafe in local area; unhappy with home environment; caregiving responsibilities; and, suboptimal physical health. Each adversity factor was dichotomised (i.e., 0 = no exposure, 1 = exposure). All were derived from #BeeWell survey data, except for FSM, neighbourhood socio-economic disadvantage, and SEN, which were drawn from linked administrative data.

The above data were used to create two continous adversity exposure scales (for LS and MH, respectively) (see Analytical Strategy). Multiple regression models containing all 14 adversity variables indicated that there was no evidence of multicollinearity. Additional, categorical versions of these scales were created to distinguish between those facing high and low levels of adversity exposure (top third and bottom third in each scale, respectively).

#### Wellbeing (T2)

Our two (LS and MH) T2 wellbeing measures were used in step 1 of our analysis to derive the resilience metric using regression residuals, and in step 2 to assess the effects of candidate protective factors. The Office for National Statistics LS item (“Overall, how satisfied are you with your life nowadays?” [44]) provides a scale from 0 (most dissatisfied) to 10 (most satisfied). The emotional difficulites subscale of the Me and My Feelings questionnaire [45] provides a measure of internalising MH difficulties, operationalised through a 0–20 scale derived from responses (Never = 0, Sometimes = 1, Always = 2) to 10 items (e.g., “I worry a lot”).

#### Resilience

A resilience metric was developed based on regression residuals using the procedures outlined below in Analytical Strategy. Values on this continuous scale range from vulnerability (negative residual scores that fall below the fitted regression line) to resilience (positive residual scores that fall above the fitted regression line).

#### Protective factors (T1)

Three external (school staff support, friendships and peer support, and family support) and five internal (sleep hygiene, physical activity, self-esteem, emotional regulation, optimism) candidate protective factors were used. For the internal factors, a distinction is drawn between those pertaining to behaviour/activity (e.g., physical activity) and those relating to psychological characteristics (e.g., optimism). All candidate protective factors were operationalised through standardised continuous scales (mean = 0, standard deviation = 1; see Table 1) with the exception of sleep hygiene, which was a dichotomous variable (0 = not getting enough sleep, 1 = getting enough sleep).

#### Covariates (T1)

T1 covariates considered in step 2 were gender (male, female), and ethnicity (categories enumerated in Table 1). For both, data were drawn from a linked administrative dataset.

### Procedure

Ethical approval from the authors’ host institution was sought and granted prior to the commencement of data collection (Ref: 2021-11133-18179). Opt-out parent/carer consent and student assent was used, leading to 1% of the overall #BeeWell sample being prevented from participation. Surveys were administered en masse to participants in school settings (typically in lessons or form time), supported by school staff (who provided standardised instructions), via a secure online survey platform (Qualtrics). Measures were presented in a random order to spread missing data due to item fatigue evenly across the survey.

### Analytic Strategy

Our analysis involved two steps. In step 1, to address RQ1, we created the T1 LS and MH adversity scales described in the previous section. These scores were summed and weighted according to their predictive utility for T2 LS and MH, which was assessed using the co-efficient effect size in a series of individual regressions with T2 LS/MH as the response variable and each adversity variable as the only explanatory variable. The results of these regressions are presented in Table 3.

**Table 3:** Individual Regressions for Life Satisfaction Adversity and Mental Health (Internalising Symptoms) Adversity

Adversity variables (T1)	LS (T2)				MH (T2)			
	Standardised β		S.E	95% C.I	Standardised β		S.E	95% C.I
Bullying victimisation	− 0.52***		0.03	[− 0.58, − 0.46]	0.66***		0.03	[0.60, 0.72]
Free school meal eligibility	− 0.16***		0.02	[− 0.20, − 0.12]	0.09***		0.02	[0.05, 0.13]
Neighbourhood socio− economic disadvantage	− 0.07**		0.02	[− 0.11, − 0.03]	0.01		0.02	[− 0.03, 0.05]
Home material deprivation	− 0.8***		0.05	[− 0.90, − 0.70]	0.56***		0.05	[0.46, 0.66]
Racial discrimination	− 0.32***		0.02	[− 0.36, − 0.28]	0.23***		0.02	[0.19, 0.27]
Gender discrimination	− 0.58***		0.02	[− 0.62, − 0.54]	0.67***		0.02	[0.63, 0.71]
Sexuality discrimination	− 0.63***		0.03	[− 0.69, − 0.57]	0.75***		0.03	[0.69, 0.81]
Disability discrimination	− 0.41***		0.03	[− 0.47, − 0.35]	0.43***		0.03	[0.37, 0.49]
Religious discrimination	− 0.27***		0.03	[− 0.33, − 0.21]	0.21***		0.03	[0.15, 0.27]
Special educational needs	− 0.05*		0.03	[− 0.11, 0.01]	0.08**		0.03	[0.02, 0.14]
Feeling Unsafe in Local Area	− 0.39***		0.04	[− 0.47, − 0.31]	0.34***		0.04	[0.26, 0.42]
Unhappy with Home Environment	− 0.81***		0.04	[− 0.89, − 0.73]	0.64***		0.04	[0.56, 0.72]
Caregiving responsibilities	− 0.04*		0.02	[− 0.08, 0.00]	− 0.02		0.02	[− 0.06, 0.02]
Suboptimal physical health	− 0.67***		0.03	[− 0.73, − 0.61]	0.68***		0.03	[0.62, 0.74]

Significance Levels: * 0.05 ** 0.01 *** 0.001. *C.I.* Confidence Interval, *LS* Life Satisfaction, *MH* Mental Health (internalising symptoms)

Next, to create the LS and MH resilience metrics, we regressed the T2 LS and MH data on to their respective T1 adversity scales. The standarized residuals from those regression models were used as the measures of resilience. For MH, the standardised residual score generated from the regression was reverse-coded so that, for both LS and MH, higher scores indicate greater resilience. In step 2, these resilience (residual) scores were treated as the outcome variable, and were regressed on the covariates and protective factors in a series of models. Model 1 (covariates only) included gender and ethnicity as the only explanatory variables in order to address RQ2. Model 2 (unique associations) included the 8 protective factors, introduced one at a time in 8 separate regression models (controlling for covariates) in order to address RQ3. In Model 3 (grouped associations), protective factors were introduced by groups in two separate models (internal and external protective factors) while controlling for covariates. In Model 4 (complete model) all protective factors and covariates were introduced simultaneously in the same model. Models 2 and 3 were considered as sensitivity analyses to assess the consistency of identified protective factors under differing model specifications. The results of Models 1 through 4 are presented in Table 4.

**Table 4:** Regression models assessing internal and external protective factors for resilience, and socio-demographic differences in resilience

	Model 1 (Covariates only)								Model 2 (Unique associations)							
	LS Resilience				MH resilience				LS resilience				MH resilience			
Covariates and protective factors (T1)	Standardised β		S.E	95% C.I	Standardised β		S.E	95% C.I	Standardised β		S.E	95% C.I	Standardised β		S.E	95% C.I
**Internal protective factors**																
Sleep hygiene									0.3***		0.02	[0.260.34]	0.26***		0.02	[0.22, 0.30]
Physical activity									0.06***		0.01	[0.040.08]	0.07***		0.01	[0.05, 0.09]
Self-esteem									0.22***		0.01	[0.200.24]	0.2***		0.01	[0.18, 0.22]
Emotional regulation									0.19***		0.01	[0.170.21]	0.2***		0.01	[0.18, 0.22]
Optimism									0.23***		0.01	[0.210.25]	0.18***		0.01	[0.16, 0.20]
**External protective factors**																
School staff support									0.14***		0.01	[0.120.16]	0.05***		0.01	[0.03, 0.07]
Friendships and social support									0.15***		0.01	[0.130.17]	0.14***		0.01	[0.12, 0.16]
Parents/carers support									0.13***		0.01	[0.110.15]	0.05***		0.01	[0.03, 0.07]
**Covariates**																
Gender (ref.: male)																
*Female*	***− 0.33		0.02	[− 0.37, − 0.29]	− 0.59***		0.02	[− 0.63− 0.55]	− 0.24***/− 0.33***		*0.02*	[− 0.28/− 0.37− 0.20/− 0.29]	0.50***/0.58***		*0.02*	[0.46/0.540.54/0.62]
Ethnicity (ref.: White)																
*Black*	0.09		0.05	[− 0.0, 10.19]	0.32***		0.05	[0.220.42]	0.00/0.10*		*0.05*	[− 0.10/0.00, 0.10/0.20]	− 0.26***/− 0.33***		*0.05*	[− 0.36/− 0.43, − 0.16/− 0.23]
*Asian*	0.13***		0.03	[0.07, 0.19]	0.26***		0.03	[0.200.32]	0.07**/0.14***		*0.03*	[0.01/0.08, 0.13/0.20]	− 0.21***/− 0.28***		*0.03*	[− 0.27/− 0.34, − 0.15/− 0.22]
*Chinese*	0.04		0.1	[− 0.16, 0.24]	0.13		0.09	[− 0.050.31]	*0.00/0.12*		*0.10*	[− 0.20/− 0.08, 0.20/0.32]	− 0.10/− 0.17*		*0.09*	[− 0.28/− 0.35, − 0.08/− 0.01]
*Any other ethnic group*	0.14*		0.07	[0.00, 0.28]	0.12		0.06	[0.000.24]	0.09/0.17*		*0.07*	[− 0.05/0.03, 0.23/0.31]	− 0.08/− 0.14*		*0.06*	[− 0.04/− 0.02, − 0.20/− 0.26
*Mixed race*	0.02		0.04	[− 0.06, 0.10]	0.18***		0.04	[0.100.26]	*0.01/0.05*		*0.04*	[− 0.07/− 0.03, 0.09/0.13]	− 0.18***/− 0.19***		*0.04*	[− 0.26/− 0.27, − 0.10/− 0.11]
*Unclassified*	0.1		0.07	[− 0.04, 0.24]	0.13		0.07	[− 0.010.27]	*0.06/0.10*		*0.07*	[− 0.08/− 0.04, 0.20/0.24]	− 0.14/− 0.14*		*0.07*	[− 0.28/− 0.28, − 000./− 0.00]

	Model 3 (Grouped associations)								Model 4 (Complete model)							
	LS resilience				MH resilience				LS resilience				MH resilience			
Covariates and protective factors (T1)	Standardised β		S.E	95% C.I	Standardised β		S.E	95% C.I	Standardised β		S.E	95% C.I	Standardised β		S.E	95% C.I
**Internal protective factors**																
Sleep hygiene	0.08***		0.02	[0.04, 0.12]	0.06**		0.02	[0.02, 0.10]	0.07**		0.02	[0.03, 0.11]	0.08***		0.02	[0.04, 0.12]
Physical activity	0.01		0.01	[− 0.01, 0.03]	0.03**		0.01	[0.01, 0.05]	0.01		0.01	[− 0.01, 0.03]	0.03**		0.01	[0.0, 10.05]
Self− esteem	0.1***		0.01	[0.08, 0.12]	0.09***		0.01	[0.07, 0.11]	0.09***		0.01	[0.07, 0.11]	0.09***		0.01	[0.07, 0.11]
Emotional regulation	0.08***		0.01	[0.06, 0.10]	0.12***		0.01	[0.10, 0.14]	0.08***		0.01	[0.06, 0.10]	0.13***		0.01	[0.11, 0.15]
Optimism	0.12***		0.01	[0.10, 0.14]	0.06***		0.01	[0.04, 0.08]	0.11***		0.01	[0.09, 0.13]	0.08***		0.01	[0.06, 0.10]
**External protective factors**																
School staff support	0.07***		0.01	[0.05, 0.09]	0.01		0.01	[− 0.01, 0.03]	0.02		0.01	[0.00, 0.04]	− 0.04***		0.01	[− 0.06, − 0.02]
Friendships and social support	0.1***		0.01	[0.08, 0.12]	0.14***		0.01	[0.12, 0.16]	0.01		0.01	[− 0.01, 0.03]	0.04***		0.01	[0.02, 0.06]
Parents/carers support	0.06***		0.01	[0.04, 0.08]	0.01		0.01	[− 0.01, 0.03]	0.01		0.01	[− 0.01, 0.03]	− 0.07***		0.01	[− 0.09, − 0.05]
**Covariates**																
Gender (ref.: male)																
*Female*	− 0.20***/− 0.31***		*0.02*	[− 0.24/− 0.35, − 0.16/− 0.27]	0.46***/0.58***		*0.02*	[0.42/0.54, 0.50/0.62]	− 0.2***		0.02	[− 0.24, − 0.16]	− 0.46***		0.01	[− 0.48, − 0.44]
Ethnicity (ref.: white)																
*Black*	*− 0.01/0.09*		*0.05*	[− 0.11/− 0.01, 0.09/0.19]	− 0.25***/− 0.32***		*0.05*	[− 0.35/− 0.42, − 0.15/− 0.22]	0.00		0.05	[− 0.10, 0.10]	0.24***		0.04	[0.16, 0.32]
*Asian*	0.06*/0.11***		*0.03*	[0.00/0.05, 0.12/0.17]	− 0.20***/− 0.24***		*0.03*	[− 0.26/− 0.30, − 0.14/− 0.18]	0.06*		0.03	[0.00, 0.12]	0.19***		0.02	[0.15, 0.23]
*Chinese*	*0.07/0.10*		*0.10*	[− 0.13/− 0.10, 0.27/0.30]	*− 0.15/− 0.14*		*0.09*	[− 0.33/− 0.32, − 0.03/− 0.04]	0.08		0.09	[− 0.10, 0.26]	0.12		0.09	[− 0.06, 0.30]
*Any other ethnic group*	0.08/0.15*		*0.07*	[− 0.06/0.01, 0.22/0.29]	*− 0.08/− 0.12*		*0.06*	[− 0.20/− 0.24, − 0.04/0.00]	0.09		0.06	[− 0.03, 0.21]	0.06		0.06	[− 0.06, 0.18]
*Mixed race*	*0.01/0.04*		*0.04*	[− 0.07/− 0.04, 0.09/0.12]	− 0.18***/− 0.18***		*0.04*	[− 0.26/− 0.26, − 0.10/− 0.10]	0.02		0.04	[− 0.06, 0.10]	0.16***		0.04	[0.08, 0.24]
*Unclassified*	*0.06/0.09*		*0.07*	[− 0.08/− 0.05, 0.20/0.23]	− 0.11/− 0.13*		*0.07*	[− 0.25/− 0.27, − 0.03/0.01]	0.04		0.07	[− 0.10, 0.18]	0.11		0.06	[− 0.01, 0.23]

Significance Levels: * 0.05 ** 0.01 *** 0.001. For covariates in unique associations models (Model 2) and grouped associations models (Model 3), the range of estimates across models is reported in italics. *C.I.* Confidence Interval, *LS* Life satisfaction, *MH* Mental health (internalising symptoms). T1 indicates that the data were collected in the first year of the #BeeWell study (Autumn 2021), when participants were 12–13 years old. T2 indicates that the data were collected in the second year of the #BeeWell study (Autumn 2022), when participants were 13–14 years old

Following the above, several additional regression models were fitted to address RQ4. These models are presented in Table 5. First, to assess gender differences in the effect of protective factors, Model 5 (unique associations by gender) included as explanatory variables the interaction between gender and each of the 8 protective factors, introduced one at a time in 8 separate regression models that controlled for the other covariate (ethnicity). Second, to assess differences in the effect of protective factors by the level of adversity exposure, Model 6 (unique associations by the level of adversity exposure) included as explanatory variables the interaction between the categorical adversity variable (high vs low) and the 8 protective factors, introduced one at a time in 8 separate regression models that controlled for the covariates gender and ethnicity.

**Table 5:** Regression models assessing internal and external protective factors for resilience, by gender and level of adversity

Protective factors (t1)	Model 5 (Unique Associations): differences between males and females																							
	LS Resilience												MH Resilience											
	Males				Females				Difference				Males				Females				Difference			
	Standardised β		S.E	95% C.I	Standardised β		S.E	95% C.I	Standardised β		S.E	95% C.I	Standardised β		S.E	95% C.I	Standardised β		S.E	95% C.I	Standardised β		S.E	95% C.I
**Internal factors**																								
Sleep hygiene	0.28***		0.03	[0.22, 0.34]	0.32***		0.03	[0.26, 0.38]	0.03		0.00	[0.03, 0.03]	0.24***		0.01	[0.22, 0.26]	0.27***		0.07	[0.13, 0.41]	0.04		0.04	[− 0.04, 0.12]
Physical activity	0.07***		0.01	[0.05, 0.09]	0.06***		0.01	[0.04, 0.08]	− 0.01		0.02	[− 0.05, 0.03]	0.11***		0.01	[0.09, 0.13]	0.04**		0.07	[− 0.10, 0.18]	− 0.09***		0.02	[− 0.13, − 0.05]
Self− esteem	0.20***		0.01	[0.18, 0.22]	0.24***		0.01	[0.22, 0.26]	0.04		0.03	[− 0.02, 0.10]	0.17***		0.01	[0.15, 0.19]	0.21***		0.01	[0.19, 0.23]	0.04		0.02	[0.00, 0.08]
Emotional regulation	0.18***		0.01	[0.16, 0.20]	0.21***		0.01	[0.19, 0.23]	0.03		0.02	[− 0.01, 0.07]	0.20***		0.01	[0.18, 0.22]	0.21***		0.01	[0.19, 0.23]	0.01		0.02	[− 0.03, 0.05]
Optimism	0.21***		0.01	[0.19, 0.23]	0.24***		0.01	[0.22, 0.26]	0.03		0.02	[− 0.01, 0.07]	0.16		0.01	[0.14, 0.18]	0.19***		0.01	[0.17, 0.21]	0.03		0.04	[− 0.05, 0.11]
**External factors**																								
School staff support	0.14***		0.01	[0.12, 0.16]	0.13***		0.01	[0.11, 0.15]	− 0.02		0.02	[− 0.06, 0.02]	0.05**		0.01	[0.03, 0.07]	0.06***		0.01	[0.04, 0.08]	0.01		0.02	[− 0.03, 0.05]
Friendships and Social Support	0.14***		0.01	[0.12, 0.16]	0.17***		0.01	[0.15, 0.19]	0.03		0.02	[− 0.01, 0.07]	0.14***		0.01	[0.12, 0.16]	0.14***		0.01	[0.12, 0.16]	0.00		0.02	[− 0.04, 0.04]
Parents/carers support	0.13***		0.01	[0.11, 0.15]	0.13***		0.01	[0.11, 0.15]	0.00		0.02	[− 0.04, 0.04]	0.06***		0.01	[0.04, 0.08]	0.04***		0.01	[0.02, 0.06]	0.02		0.02	[− 0.02, 0.06]

	Model 6 (Unique Associations): differences between adolescents facing low adversity and adolescents facing high adversity																							
	LS resilience												MH resilience											
	Males				Females				Difference				Males				Females				Difference			
Protective factors (t1)	Standardised β		S.E	95% C.I	Standardised β		S.E	95% C.I	Standardised β		S.E	95% C.I	Standardised β		S.E	95% C.I	Standardised β		S.E	95% C.I	Standardised β		S.E	95% C.I
**Internal factors**																								
Sleep hygiene	0.38***		0.04	[0.30, 0.46]	0.25***		0.04	[0.17, 0.33]	− 0.12*		0.05	[− 0.22, − 0.02]	0.34***		0.03	[0.28, 0.40]	0.21***		0.04	[0.13, 0.29]	− 0.11*		0.05	[− 0.21, − 0.01]
Physical activity	0.10***		0.02	[0.06, 0.14]	0.03		0.02	[− 0.01, 0.07]	− 0.06*		0.03	[− 0.12, 0.00]	0.09***		0.02	[0.05, 0.13]	0.05**		0.02	[0.01, 0.09]	− 0.03		0.02	[− 0.07, 0.01]
Self-esteem	0.28***		0.02	[0.24, 0.32]	0.21***		0.02	[0.17, 0.25]	− 0.07*		0.03	[− 0.13, − 0.01]	0.26***		0.03	[0.20, 0.32]	0.18***		0.02	[0.14, 0.22]	− 0.07*		0.03	[− 0.13, − 0.01]
Emotional regulation	0.23***		0.03	[0.17, 0.29]	0.18***		0.02	[0.14, 0.22]	− 0.04		0.03	[− 0.10, 0.02]	0.25***		0.02	[0.21, 0.29]	0.18***		0.02	[0.14, 0.22]	− 0.05*		0.03	[− 0.11, 0.01]
Optimism	0.28***		0.02	[0.24, 0.32]	0.21***		0.02	[0.17, 0.25]	− 0.07*		0.03	[− 0.13, − 0.01]	0.22***		0.02	[0.18, 0.26]	0.14***		0.02	[0.10, 0.18]	− 0.06*		0.03	[− 0.12, 0.00]
**External factors**																								
School staff support	0.20***		0.02	[0.16, 0.24]	0.09***		0.02	[0.05, 0.13]	− 0.11***		0.03	[− 0.17, − 0.05]	0.11***		0.02	[0.07, 0.15]	0.00		0.02	[− 0.04, 0.04]	− 0.11***		0.01	[− 0.13, − 0.09]
Friendships and Social Support	0.25***		0.02	[0.21, 0.29]	0.10***		0.02	[0.06, 0.14]	− 0.15***		0.03	[− 0.21, − 0.09]	0.21***		0.02	[0.17, 0.25]	0.08***		0.02	[0.04, 0.12]	− 0.13***		0.01	[− 0.15, − 0.11]
Parents/carers support	0.22***		0.02	[0.18, 0.26]	0.08***		0.02	[0.04, 0.12]	− 0.14**		0.03	[− 0.20, − 0.08]	0.13***		0.02	[0.09, 0.17]	0.00		0.02	[− 0.04, 0.04]	− 0.13***		0.03	[− 0.19, − 0.07]

Significance Levels: * 0.05 ** 0.01 *** 0.001. *C.I.* Confidence Interval, *LS* Life satisfaction; MH = Mental health (internalising symptoms). T1 indicates that the data were collected in the first year of the #BeeWell study (Autumn 2021), when participants were 12–13 years old. T2 indicates that the data were collected in the second year of the #BeeWell study (Autumn 2022), when participants were 13–14 years old. Low adversity = Bottom third of the adversity scale; High adversity = Top third of the adversity scale

To account for missing data (see levels of missing data in the first column of Table 1), multiple imputation was used for all the study variables with missing data. We performed 20 imputations of the data set using multivariate normal regression approach. All analyses were conducted in STATA 15 [52].

## Results

### Adversity exposure and later wellbeing (RQ1)

Table 3 shows the results of the individual regressions to estimate the relative importance of each adversity variable to the two wellbeing outcomes. For LS, effect sizes ranged from –0.04 standard deviations (S.D.) (caregiving responsibilities) to – 0.80 S.D. (home material deprivation). For MH, effect sizes ranged from 0.08 S.D. (SEN) to 0.75 S.D. (sexuality discrimination). T1 neighbourhood socio-economic disadvantage and caregiving responsibilities were not statistically significant predictors of T2 MH. As a result, two distinct adversity indices were created as described in the previous section, for LS (min = 0, max = 5.71, mean = 0.77) and MH (min = 0, max = 5.32, mean = 0.73) respectively.

### Resilience: socio-demographic differences across gender and ethnicity (RQ2)

Model 1 in Table 4 reveals gender differences in resilience. Specifically, girls are significantly less resilient than boys for both wellbeing outcomes (standardised effect size for LS − 0.33; for MH − 0.59). This is also evident in the complete model (Model 4), though the differences attenuate somewhat (for LS − 0.20; for MH − 0.46), indicating that gender disparities in protective factors explain part of the observed inequalities in resilience.

Ethnic differences in resilience are also observed in Table 4. Model 1 reveals that, compared to white adolescents, Asian and 'any other ethnic group' adolescents are significantly more resilient in terms of LS (0.13 and 0.14, respectively). In terms of MH resilience, compared to white adolescents, those of black, Asian and mixed race are significantly more resilient (0.32, 0.26 and 0.18, respectively). In the LS complete model (Model 4), the resilience gap between white and 'any other ethnic group' adolescents disappears, and the disparity between white and Asian adolescents, while still statistically significant, attenuates (0.06). In terms of the MH complete model (Model 4), all ethnic differences noted above remain statistically significant but shrink (white vs black 0.23; white vs Asian 0.19; white vs mixed race 0.16). As above, this indicates that disparities in protective factors explain part of the observed ethnic inequalities in resilience.

### Protective factors for resilience (RQ3)

Table 4 shows that for all the internal and external factors studied, there is evidence of protective effects in one or several of the models. However, differences across model specifications are substantial in some cases.

Sleep hygiene is the only binary protective factor studied. Accordingly, effect sizes for this variable cannot be directly compared to those of the other (continuous) protective factors noted below. Model 2 (unique associations) shows that adolescents getting enough sleep show significantly higher LS and MH resilience than those who do not (for LS 0.30, for MH 0.26). When controlling for other internal protective factors in Model 3 (grouped associations), the protective effect of sleep hygiene is still evident but attenuates (for LS 0.08; for MH 0.06); this pattern is also evident when also controlling for external factors in the complete model (Model 4; for LS 0.07, for MH 0.08).

In Model 2 (unique associations), adolescents who are more physically active exhibit significantly higher resilience for both wellbeing outcomes (for LS 0.06, for MH 0.07). When controlling for other internal protective factors in Model 3 (grouped associations), the protective effect disappears for LS resilience, and reduces but remains statistically significant for MH resilience (0.03); this pattern is also evident when also controlling for external factors in the complete model (Model 4; no effect for LS, for MH 0.03).

In terms of the three internal psychological factors, the unique associations model (Model 2) reveals significant protective effects for each (self-esteem: for LS 0.22, for MH 0.20; emotional regulation: for LS 0.19, for MH 0.20; optimism: for LS 0.23, for MH 0.18). When controlling for other internal protective factors in the grouped associations model (Model 3), these protective effects reduce but remain statistically significant (self-esteem: for LS 0.10, for MH 0.09; emotional regulation: for LS 0.08, for MH 0.12; optimism: for LS 0.12, for MH 0.06); this pattern is also evident when also controlling for external factors in the Model 4 (self-esteem: for LS 0.09, for MH 0.09; emotional regulation: for LS 0.08, for MH 0.13; optimism: for LS 0.11, for MH 0.08).

With regard to the three external factors examined, the unique associations model (Model 2), reveals significant protective effects for each (school staff support: for LS 0.14, for MH 0.05; friendships and social support: for LS 0.15, for MH 0.14; family support: for LS 0.13, for MH 0.05). When controlling for other external protective factors in the grouped associations model (Model 3), these protective effects for LS resilience reduce but remain statistically significant (school staff support 0.07; family support: 0.06; friendships and social support: 0.10). In terms of MH resilience in Model 3, the protective effect remains the same for friendships and social support (0.14) but disappears for both school staff support and family support. When also controlling for internal protective factors in the complete model (Model 4), protective effects for LS resilience disappear for all three external factors. By contrast, protective effects for MH resilience reduce but remain statistically significant for friendships and social support (0.05), and *reverse* for school staff support (– 0.04) and family support (– 0.06).

### Protective factors for resilience: differences by gender and level of adversity (RQ4)

In relation to gender differences in protective factors for resilience, Table 5 shows that there are no statistically significant differences between girls and boys in the protective effect of candidate factors for LS resilience. However, in terms of MH resilience, physical activity yields a stronger protective effect for boys than for girls (diff. – 0.09).

Table 5 also shows a clear pattern in which the protective factors studied have a stronger effect for those at lower levels of adversity exposure than for those at higher levels. In terms of LS resilience, this pattern is observed for all factors except emotional regulation (sleep hygiene diff. – 0.12; physical activity diff. – 0.06; self-esteem diff. − 0.07; optimism diff. – 0.07; school staff support diff. – 0.11; friendships and social support diff. – 0.15; family support diff. – 0.14). In terms of MH resilience, the pattern is observed for all factors except physical activity (sleep hygiene diff. – 0.11; self-esteem diff. – 0.07; emotional regulation diff. – 0.05; optimism diff. – 0.06; school staff support diff. – 0.11; friendships and social support diff. – 0.13; family support diff. – 0.13).

## Discussion

In the current study, we sought to extend understanding of the interplay between exposure to adversity and access to protective factors in determining wellbeing outcomes during a particularly vulnerable period of adolescent development. Novelty and rigour are offered through our focus on the transition from early to middle adolescence (age 12–14); use of the residuals analytical approach; analysis of a large, longitudinal dataset, collected post-Covid-19; inclusion of a broad range of adversity and protective factors; consideration of socio-demographic disparities in protective factors and resilience; and, examination of two key wellbeing outcomes: life satisfaction (LS) and internalising mental health (MH) symptoms.

### Adversity exposure and later wellbeing (RQ 1)

Consistent with resilience theory [2] and prior research [53], a range of adversity factors operating at multiple systemic levels predicted later reductions in wellbeing. Notably, being unhappy with home environment, home material deprivation, sexuality discrimination, suboptimal physical health, gender discrimination, and bullying were each associated with > 0.5 SD change in both later wellbeing outcomes. These findings offer valuable new insights as they emphasize the importance of certain harmful or abusive relationships, interactions, and experiences which are often neglected in research on child and adolescent adversity exposure (e.g., fewer than 10% of studies in Hughes et al. meta-analysis [54] of adverse childhood experiences on health outcomes included bullying, and no studies included experiences of discrimination).

Though many adversity factors were common to both outcomes, there was also clear evidence of differentiation for some, with disparities in relative magnitude (e.g. bullying more strongly associated with later MH than LS; contrastingly, home material deprivation more strongly associated with later LS than MH). Furthermore, two adversity variables (neighbourhood socio-economic disadvantage and caregiving responsibilities) predicted later LS but *not* MH. This pattern of findings is broadly consistent with the aforementioned characterisation of adversity factors representing threat and deprivation respectively, and the proposition that they result in distinct downstream consequences for later outcomes [4]. For example, it is noteworthy that threat factors (e.g. sexuality discrimination, gender discrimination, bullying) were consistently associated with larger changes in later MH than LS.

### Socio-demographic differences in resilience (RQ2)

Girls displayed significantly lower levels of resilience than boys, particularly with regard to MH. This is consistent with previous findings focusing on gender differences in resilience among particular population subgroups (e.g. burns patients [55]; those affected by natural disasters [56]). However, studies on adolescence resilience using the residuals approach have so far provided mixed evidence [22, 23, 27, 32]. These contrasting findings may be the result of differences in the age group, wellbeing measure, and adversity factors considered in these studies, as well as further contextual considerations. Taken alongside consistent evidence of a significantly higher propensity for internalising MH symptoms and lower LS among girls during adolescence [57, 58], these findings prompt the need for further consideration of gender-specific resilience-related processes [59].

Turning to ethnicity, white adolescents displayed significantly lower levels of resilience than some minority ethnic groups; as above, this was particularly apparent with regard to MH. To our knowledge, the only adolescent residuals study to have considered ethnic differences in resilience is Cahill et al. [23], which found no evidence of ethnic differences using a binary approach to coding ethnicity data (i.e., White vs. ethnic minority). It is possible that these discrepancies may be explained by differences in the approach to categorise ethnic groups in the UK, as the approach in Cahill et al. [23] may have masked important differences across discrete ethnic minority groups. There is some evidence that young people from UK minority ethnic groups have similar or better MH than their white peers [60, 61], but findings are inconsistent [62]. However, there is more consistent evidence that Asian adolescents present better MH [63, 64] and higher subjective well-being [65] than white adolescents, which aligns with our findings (since their significantly higher levels of resilience identified here would predict comparable or better wellbeing *outcomes*). Furthermore, although research on ethnic differences in adolescent resilience in the UK is relatively scarce, our findings are in line with recent evidence indicating that adolescents from some ethnic minority groups displayed better MH adaptation to the covid-19 pandemic than white adolescents [66, 67].

Collectively, our analyses provide evidence that gender and ethnicity may be important moderators in the processes through which exposure to adversity and access to protective factors determine wellbeing outcomes. Further research is required to better understand *why* this is the case.

### Protective factors for resilience (RQ3)

Evidence of the protective effects of all internal and external factors studied was found. However, protective effects attenuated (and in some cases disappeared) in the grouped associations and complete models, indicating some sensitivity to model specification. This was particularly evident in the case of external factors (e.g., school staff support). Nonetheless, adolescents who reported getting enough sleep displayed higher resilience across all models and outcomes, and higher levels of physical activity consistently predicted MH resilience specifically. These findings are in line with broader research on the links between these two health-related behaviours and wellbeing [68, 69], in addition to extant theory on how they *could* promote resilience during adolescence [70]. However, ours is the first study to empirically establish these protective effects. Sleep hygiene and physical activity offer particular promise as modifiable protective factors given their obvious tractability, with robust meta-analytic evidence of meaningful intervention effects in both cases [71, 72]. However, further work is required to consider effective intervention design for the *adolescent* population if the full potential of these health-related behaviours to promote resilience is to be realised.

Internal psychological factors (self-esteem, emotional regulation, and optimism) yielded some of the strongest and most consistent (across outcomes and specifications) protective effects in our models. With reference to self-esteem, our findings mirror those of other residuals studies of children and adolescents, namely Cahill et al. [23], and Miller-Lewis et al. [22] (albeit the latter study identified protective effects for the related construct of self-concept rather than self-esteem specifically), and in doing so reinforce the importance of valuing oneself as a key resilience factor for young people. With regard to emotion regulation, there are parallels with Miller-Lewis et al. identification of the protective effects of self-control in their residuals study [22], and, more broadly, meta-analytic research findings which demonstrate moderate associations between emotion regulation strategies and mental health [73, 74]. Finally, set against the backdrop of no residuals studies and limited empirical scrutiny of genuine protective effects using other approaches [75], our identification of optimism as a protective factor here offers important new evidence of the salience of engendering positive expectations about the future in helping adolescents be more resilient to the effects of current adversities.

The three external factors examined (school staff support, friendships and social support, and parents/carers support) generally yielded smaller and more variable (across outcomes and specifications) protective effects than the internal factors noted above. However, with one exception (complete model for LS), there was consistent evidence of friendships and social support as a protective factor for resilience. These findings mirror those of Van Harmelen et al. [27] aforementioned residuals study, and are supported by broader, meta-analytic evidence focusing on the association between peer social support and wellbeing in adolescence [76, 77]. By contrast, the protective effects of school staff support were smaller and more sensitive to model specification. This is somewhat consistent with Miller-Lewis et al. [22] finding, established through interaction effects as opposed to the residuals approach, of a very small protective effect of child-teacher relationships that varied by model. Interestingly, Cahill et al. residuals analysis identified protective effects of *positive perceptions* of school [23], and there is meta-analytic research evidence of the association between school *connectedness* and some aspects of adolescent wellbeing [78]. These findings indicate that young people’s more general sense of attachment to school may be more important than the quality of their specific relationships with staff in conferring resilience. More broadly, the discrepancies speak to the ‘two worlds’ hypothesis [79, 80], which argues that young people would perceive their school life as involving two aspects rather than just one (learning-related aspects such as grades, attainment and relationships with school staff; and classmates-related aspects, such as bullying and relationships with peers). Evidence that classmates-related aspects tend to be more important to adolescents’ wellbeing than learning-related aspects [80], alongside the contrasting findings noted above, prompts further empirical scrutiny of what specific aspects of school-related experiences may yield protective effects.

Finally, parent/carer support yielded the smallest and least consistent protective effects overall. Indeed, it is perhaps noteworthy that in the final, full model, the effect of this factor (and that of school staff support) actually *reversed* (i.e., predicted *lower* levels of MH resilience). While this could be a statistical artefact (e.g., overfitting), there are interesting parallels with Van Harmelen’s aforementioned residuals study with adolescents [27], which similarly identified family support as a *negative* predictor of later resilient functioning. Like these authors, we are left to speculate that family involvement may not be adaptive in the context of adversity (which may, of course, include familial adversity factors). One reason why support from school staff and/or parents/carers could confer limited protection against adversity is that those adolescents who are more resilient are developing higher autonomy (i.e., lower dependency on this type of support), as might be expected in this developmental phase. Thus, we might hypothesize that school staff and parent/carer support would yield greater protective effects during *childhood* as opposed to adolescence. Further research is required to explore this issue in more detail. Collectively, though, our findings speak to the clear salience of peer social relations as conferring protection in the context of multiple adversities during the transition from early- to mid-adolescence.

### Differences in the effects of protective factors by gender and level of adversity (RQ4)

We observed gender differences in protective factors for MH resilience (but not LS resilience) for physical activity, which presented stronger protective effects for boys than for girls. In line with this, existing evidence indicates that physical activity predict adolescent wellbeing, and levels tend to be higher among boys than girls [81–83]. To the best of our knowledge, this is the first to identify protective effects for MH resilience for physical activity levels.

We found a clear pattern across resilience outcomes in which protective factors yielded a stronger effect at low (compared to high) levels of adversity exposure. This may be indicative that they serve *promotive* rather than *protective* functions as they support resilience across the adversity spectrum, rather than specifically those at high adversity levels. Furthermore, these differences were more pronounced for *external* protective factors than for the *internal* protective factors. Our findings contrast sharply with Askeland et al., who found that goal orientation and self-confidence were particularly protective for Norwegian adolescents who experienced a higher number of negative life events [84], and Miller-Lewis et al., who found that greater self-control during preschool provided greater protection to children exposed to higher levels of familial adversity [22]. However, this could be explained by the conceptualisation and measurement of adversity, age of participants, and other differences between the studies. Collectively, these findings have clear implications for the design of interventions to promote resilience, as they indicate that a tailored, nuanced approach—which takes into account differing levels of adversity, as well as socio-demographic differences—may be more effective than a one-size-fits-all model. It is this issue to which we now turn.

### Implications

Our findings confer three key implications for policy and practice. First, given that the effects of protective factors appeared to diminish at higher levels of adversity exposure, prevention and intervention efforts to reduce said exposure should be prioritised. Among the most influential adversity factors identified in the current study, some reflect broader structural inequalities (e.g., home material deprivation) that would require policy/governmental intervention [85], while other represent more immediately tractable issues (e.g., bullying) that could feasibly be targeted in localised school- and/or community-based interventions, for which there is a promising evidence base [86]. However, it is important to note that the above does not mean that universal interventions would not be beneficial, as our results indicate protective effects across the adversity spectrum for all the internal factors and friendships and social support. Second, resilience can be most effectively promoted through multi-faceted school- and/or community-based interventions intended to facilitate access to protective factors identified in the current study. In this vein, we note that there is robust evidence of the efficacy of interventions to promote sleep hygiene [87], physical activity [88], self-esteem [89], emotional regulation [90], optimism [91], school staff support [92], friendships and social support [93], and parent/carer support [94] in adolescence. Third, our finding that the protective effect of these factors differs by gender and level of adversity exposure indicates that a nuanced, tailored/targeted approach, alongside a universal offer, could optimise resilience promotion.

Our study also presents valuable insights for future research. First, our findings provide support to the call by Mesman et al. [8] on the need for and value of more longitudinal research using a multisystem approach and advanced assessment methods such as the residuals approach or network modelling [95]. Second, our findings highlight the need for nuanced approaches to assessing socio-demographic differences in protective factors for resilience. Third, our study also shows the importance of assessing resilience for distinct wellbeing constructs. Finally, the current study highlights the importance of assessing a wide range of adversity factors, beyond the ‘usual suspects’ (i.e., familial adversities).

### Limitations

In considering the above implications, a number of limitations of the current study should be borne in mind, most of which pertain to the fact that we undertook secondary analysis of an existing dataset as opposed to a prospectively designed study. First, a number of key adversity factors (e.g., parental psychological distress, living in a single-parent family, household violence, etc.) were not included in our analyses. However, this is arguably counterbalanced by the inclusion of a number of new or relatively under-studied factors. Second, the analyses cover a relatively short period of time (1 year) and a limited age range (12–14). Nonetheless, this did of course enable insights into a particularly vulnerable period of adolescent development.

Third, while the composition of the study sample reflected that of the 11–16 population of the city-region (Greater Manchester) from which it was drawn very well, there are some noteworthy differences from the equivalent population in England (e.g., ethnic composition – somewhat higher proportion of Asian and lower proportion of white adolescents than is seen nationally) that suggest caution is required in terms of generalisation. Furthermore, we note that although complete and incomplete cases were generally very similar, the latter group were over-represented in two socioeconomic adversity indicators (FSM eligibility and neighbourhood socioeconomic deprivation) as is often observed in longitudinal studies [96]. More broadly, the study was conducted in a Western, high-income country using a population sample. Accordingly, and particularly in light of research that highlights differences in adversity and resilience processes across countries and cultures [30] that which has been reported here may not necessarily apply in other parts of the world -nor in clinical samples- where levels of exposure to adversity in the population studied may be different.

Finally, we must be mindful of the possibility of shared measurement variance due to the use of self-reported information in the assessment of both exposures and outcomes. This introduces a possible source of confounding in that an unmeasured factor could explain associations. However, it is important to note again the one-year lag between reporting of exposures and outcomes, and the fact that several exposures were *not* self-reported (e.g., FSM, neighbourhood socio-economic deprivation, special educational needs).

## Conclusion

The current study has demonstrated that the residuals approach can make a considerable contribution to our understanding of the interplay between exposure to adversity and access to protective factors in determining adolescent wellbeing outcomes. Moreover, its application provides clear implications for policy and practice, in terms of prevention (of adversity exposure) and intervention (to facilitate resilience). Finally, the current study provides further support for Masten’s [5] ordinary magic thesis. Getting enough sleep, being more physically active, experiencing support from friends, and other factors exerting protective effects for adolescents experiencing adversity epitomise the maxim that “resilience arises from ordinary resources and processes” [5].

### Supplementary Information

**Additional file 1: Table S1.** Comparison of complete and incomplete cases for socio-demographic variables, adversity factors and protective factors.

## Data Availability

Not applicable.
