# Supplementary material for: Protective factors for resilience in adolescence: analysis of a longitudinal dataset using the residuals approach
Source: Child Adolesc Psychiatry Ment Health. 2023 Dec 19;17:140. doi: 10.1186/s13034-023-00687-8 (PMC10731682; doi:10.1186/s13034-023-00687-8)
Supplement: Supplementary file 1 — Additional file 1: Table S1. Comparison of complete and incomplete cases for socio-demographic variables, adversity factors and protective factors. [file 13034_2023_687_MOESM1_ESM.docx]

| **Table S1** |  |  |
| --- | --- | --- |
| Comparison of complete and incomplete cases for socio-demographic variables, adversity factors and protective factors. | | |
|  |  |  |
|  | Complete  (T1 and T2, n=12,130) | Incomplete  (T1 only, n=8,111) |
| Socio-demographic characteristics |  |  |
| Gender |  |  |
| Male | 51.35% | 48.11% |
| Female | 48.65% | 51.89% |
| Ethnicity |  |  |
| White | 65.43% | 64.96% |
| Black | 5.27% | 5.46% |
| Asian | 17.67% | 18.08% |
| Chinese | 0.97% | 0.89% |
| Any Other Ethnic Group | 2.46% | 2.25% |
| Mixed Race | 5.93% | 6.16% |
| Unclassified | 2.27% | 2.20% |
|  |  |  |
| Adversity factors (T1) |  |  |
| Bullying Victimisation | 17.01% | 18.98% |
| Free School Meal Eligibility | 24.05% | 31.29% |
| Neighbourhood Socio-Economic Disadvantage | 24.12% | 31.13% |
| Home Material Deprivation | 3.30% | 4.16% |
| Racial Discrimination | 27.35% | 28.08% |
| Gender Discrimination | 21.10% | 22.66% |
| Sexuality Discrimination | 16.16% | 17.21% |
| Disability Discrimination | 11.97% | 13.69% |
| Religious Discrimination | 16.41% | 16.43% |
| Special Educational Needs | 14.71% | 18.46% |
| Feeling Unsafe in Local Area | 6.14% | 7.41% |
| Unhappy with Home Environment | 5.26% | 6.28% |
| Caregiving Responsibilities | 47.98% | 50.52% |
| Suboptimal Physical Health | 13.58% | 16.47% |
|  |  |  |
| Protective factors (T1) |  |  |
| Sleep Hygiene | 65.67% | 61.39% |
| Physical Activity | 354.89 | 349.32 |
| Self-Esteem | 14.84 | 14.59 |
| Emotional Regulation | 24.39 | 23.92 |
| Optimism | 12.04 | 11.86 |
| School Staff Support | 15.22 | 14.90 |
| Friendships and Social Support | 15.60 | 15.40 |
| Family Support | 17.43 | 17.07 |
